# Hydrothermal Synthesis of Nanoclusters of ZnS Comprised on Nanowires

**DOI:** 10.3390/nano3030564

**Published:** 2013-09-09

**Authors:** Zafar Hussain Ibupoto, Kimleang Khun, Xianjie Liu, Magnus Willander

**Affiliations:** 1 Physical Electronics and Nanotechnology Division, Department of Science and Technology, Campus Norrköping, Linköping University, SE-60174 Norrköping, Sweden; E-Mails: kimleang.khun@liu.se (K.K.); magnus.willander@liu.se (M.W.); 2 Department of Physics, Chemistry, and Biology (IFM), Linköping University, 58183 Linköping, Sweden; E-Mail: xjliu@ifm.liu.se

**Keywords:** ZnS, nanoclusters, nanowires, hydrothermal method, CTAB

## Abstract

Cetyltrimethyl ammonium bromide cationic (CTAB) surfactant was used as template for the synthesis of nanoclusters of ZnS composed of nanowires, by hydrothermal method. The structural and morphological studies were performed by using X-ray diffraction (XRD), scanning electron microscopy (SEM) and high resolution transmission electron microscopy (HRTEM) techniques. The synthesized ZnS nanoclusters are composed of nanowires and high yield on the substrate was observed. The ZnS nanocrystalline consists of hexagonal phase and polycrystalline in nature. The chemical composition of ZnS nanoclusters composed of nanowires was studied by X-ray photo electron microscopy (XPS). This investigation has shown that the ZnS nanoclusters are composed of Zn and S atoms.

## 1. Introduction

Among II-VI compound semiconductors, zinc sulphide (ZnS) is well known due to its wide band gap of 3.7 eV at room temperature and high exciton binding energy of 40 meV. ZnS is used widely in various applications such as ultraviolet light emitting diodes [1], electroluminescence devices [2], infrared windows [3], and flat panel displays [4]. Recently, extensive efforts have been taken for the synthesis of various ZnS morphologies such as nanoparticles [5], nanorods [6], nanobelts [7,8], nanotubes [9], nanosheets [10], well aligned tetrapods [11], nanowires bundles [12,13], and hollow spheres [14,15,16,17]. Due to high surface to volume ratio of one dimensional nanomaterial, which has shown the better performance in the development of devices, more attention is paid to the synthesis of these nanostructures. Different synthetic approaches have been used for the synthesis of ZnS nanomaterial, especially nanowires and nanorods; these methods include laser ablation [18], vapor transport in the presence [19,20,21,22,23] or in the absence of [13,24] catalyst, carbothermal chemical vapor deposition [25], electrochemical deposition [26,27,28] and solvothermal method [29,30,31]. Among these methods, the hydrothermal method is preferable due to its simplicity, cost effectiveness, high yield and to achieve a controlled morphology [32,33,34]. ZnS is found more thermodynamically stable in the cubic sphalerite, however hexagonal wurtzite is thermodynamically metastable under the specific conditions and it is possible to convert hexagonal structure into a cubic crystal structure spontaneously by interacting with the organic substances [35]. ZnS is produced in a variety of nanostructures and morphologies with high chemical and thermal stabilities. For control and improvement in the dimension of nanostructures of desired product, organic solvents or additives are largely utilized in the hydrothermal growth method.

In this study, ZnS nanowires were synthesized by using cetyltrimethyl ammonium bromide cationic (CTAB) surfactant as a template for the growth. The effect of CTAB on the formation of nanowires was also investigated. Zinc acetate dihydrate was used as zinc source and thio urea as sulpher source, and CTAB as template for the growth. Scanning electron microscopy (SEM), X-ray diffraction (XRD) and transmission electron microscopy (TEM) were used for the structural characterization of prepared sample of ZnS nanowires. X-ray photoelectron spectroscopy (XPS) was used for the study of chemical composition.

## 2. Results and Discussion

**Figure 1:**
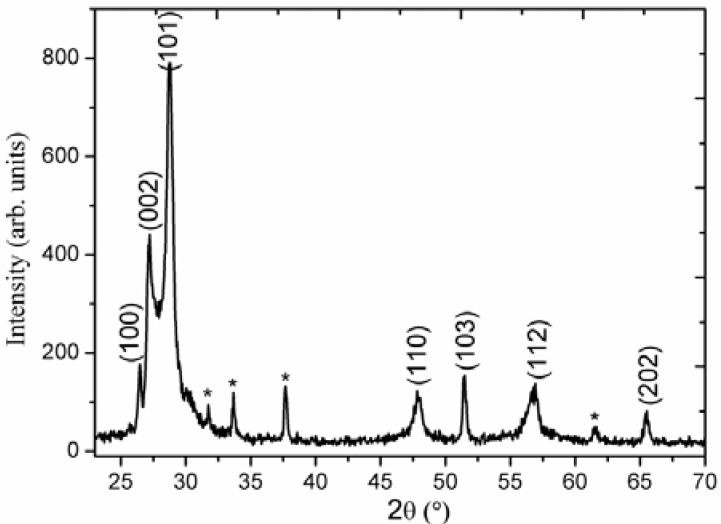
X-ray diffraction (XRD) of ZnS nanoclusters comprised on nanowires.

Figure 1 shows the XRD pattern study of ZnS nanoclusters composed of nanowires. The diffraction peaks such as 100, 002, 101, 110, 103, 112, and 202 appeared in the ZnS sample which is in accordance with the hexagonal phase of ZnS and supported by the reported work [36]. The diffraction peaks, indicated by stars in the XRD spectrum, are indexed to the fluorine doped tin oxide (FTO) glass substrate. Moreover, the peaks correspond to the polycrystalline ZnS which are also supported by [37] XRD study has shown the pure polycrystalline phase of ZnS.

**Figure 2:**
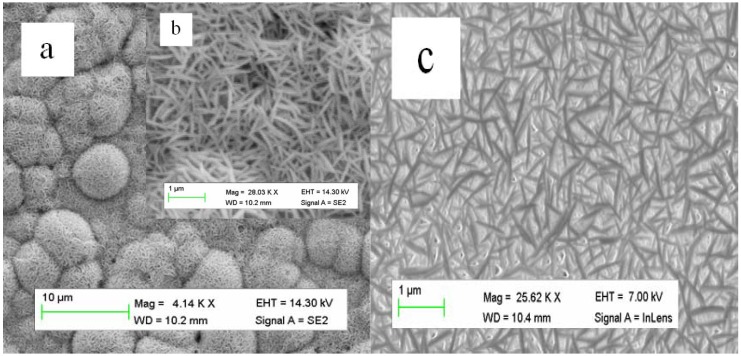
Scanning electron microscopy (SEM) images of ZnS nanomaterial: (**a**) low magnification, (**b**) high magnification, (**c**) effect of higher concentration of cetyltrimethyl ammonium bromide cationic (CTAB).

Typical SEM images of ZnS are shown in Figure 2 and it can be seen that, at low magnification, ZnS morphology is composed of nanoclusters (Figure 2a). However, at high magnification it can be observed that ZnS nanoclusters are composed of nanowires as shown in Figure 2b. We have also observed the effect of CTAB concentration on the morphology of ZnS. Figure 2c shows that with the increase of 1 g of CTAB, the nanowires tend to become thinner and smaller in length. On increasing the concentration of CTAB results in a decrease in length of ZnS nanowires which could be due to the restriction of lateral growth of ZnS nanowires. This effect, of CTAB at higher concentrations for other metal oxides, has been reported in the literature [38,39]. Moreover, as CATB is a cationic surfactant, sulpher radicals are aggregated on the surface of CTAB and result in nanoclusters of ZnS.

**Figure 3:**
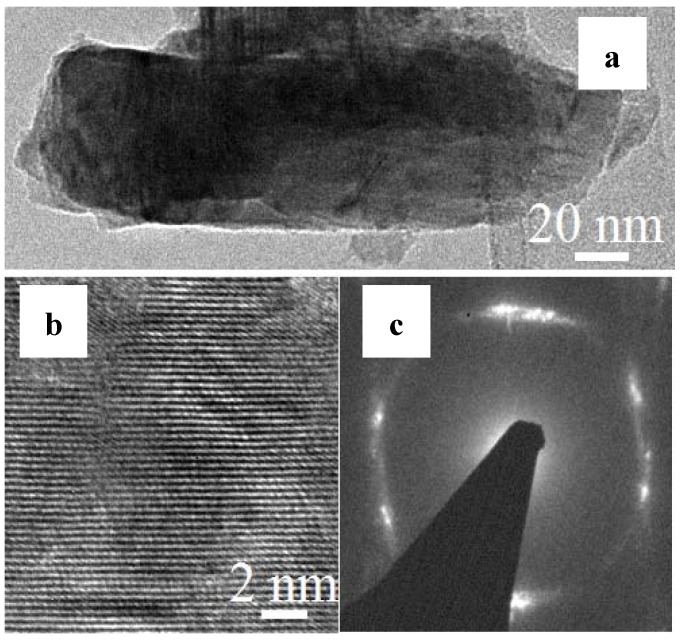
The transmission electron microscopy (TEM) study of ZnS nanoclusters: (**a**) TEM, (**b**) high resolution transmission electron microscopy (HRTEM), (**c**) selected area electron diffraction (SAED).

Distinctive TEM, high resolution transmission electron microscopy (HRTEM) and selected area electron diffraction (SAED) images are shown in Figure 3. Figure 3a is a TEM image of a ZnS single nanowire and is 5 to 10 µm in length. The HRTEM image of synthesized ZnS nanowire is shown in Figure 3b and the interplanar distance is 0.313 nm which is attributed to (002) plane of the hexagonal phase of ZnS. The SAED image is shown in Figure 3c which shows that ZnS is polycrystalline in nature.

The growth mechanism of ZnS nanoclusters composed of nanowires by hydrothermal growth method could be explained in terms of liquid-solid mechanism. By using CTAB as a growth template, it increases the concentration of sulpher free radicals in the growth solution during the heating of precursors in the Teflon vessel. The released free S radicals then might combine with the Zn^2+^ ions and result in the ZnS nanomaterial. The possible reaction involved in the formation of nanoclusters of ZnS includes:

Zn^2+^ + S·+ e^−^ → ZnS (1)

XPS analysis was carried out for the study of chemical composition and purity of prepared sample of ZnS nanoclusters.

**Figure 4:**
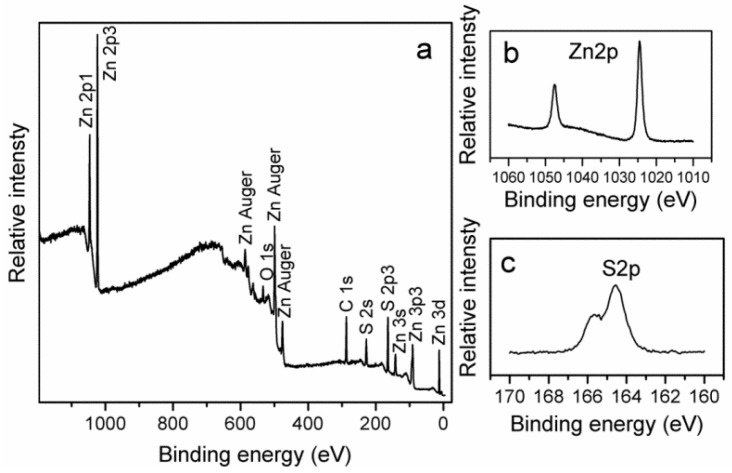
The XPS analysis of ZnS nanostructures (**a**) Core shell scan XPS spectrum of sample, (**b**) Zn2p XPS spectrum, (**c**) S2p XPS spectrum.

The XPS spectra of ZnS nanoclusters are shown in Figure 4. The scan survey spectrum of ZnS nanoclusters is shown in Figure 4a. The peaks of Zn and S are dominant, however, peaks of C and O also appear in the survey spectrum of ZnS nanoclusters. The appearance of O1s signal could be indexed to lattice oxygen (OL) of ZnO, surface hydroxyl oxygen (OH) and the chemisorbed oxygen (OA) [40]. Figure 4b shows the valence band XPS spectrum of ZnS and Figure 4c shows the binding energies of Zn 2p 1/2 and 2p 3/2 of the prepared sample of ZnS and observed at 1045.0 and 1022.1 eV respectively. For S 2p 1/2 the binding energy is observed at 162.0 eV and the appearance of all the binding energy values for Zn 2p and S 2p are in accordance with previously reported work [41], as shown in Figure 4d.

## 3. Experimental Section

The commercially available fluorine doped tin oxide (FTO) glass substrate was purchased from Sigma Aldrich Sweden and was used for the growth of ZnS nanostructures. The growth methodology was based on the following steps. Firstly, FTO glass substrate was washed with isopropanol in ultrasonic bath for 5 to 10 min and dried at room temperature. The growth solution of 0.5 M zinc acetate dihydrate and 0.75 M thio urea was prepared in 50 mL deionized water using 0.5 g and 1 g of CTAB respectively as a template for the growth of ZnS. The cleaned FTO glass substrate was affixed in the Teflon sample holder and growth solution was poured into Teflon vessel of 125 mL capacity and the Teflon vessel containing FTO substrate was sealed in the stainless steel autoclave and kept at 200 °C in the preheated electric oven for 12 h.

The characterization of ZnS nanostructures was performed by using SEM, XRD and HRTEM techniques. XPS technique was used to study the chemical composition of the prepared sample of ZnS.

## 4. Conclusions

In this study, the hydrothermal method was used for the synthesis of ZnS nanoclusters and the investigation of morphology further showed that the nanoclusters are composed of nanowires using CTAB as the template for growth. Structural, morphological and chemical composition studies were conducted. The nanoclusters of ZnS composed of nanowires are reported for the first time by hydrothermal method in this study. The effect of the CTAB concentration on the morphology of ZnS nanostructures was also investigated.
